# Dopamine-mediated striatal activity and function is enhanced in GlyRα2 knockout animals

**DOI:** 10.1016/j.isci.2023.107400

**Published:** 2023-07-17

**Authors:** Jens Devoght, Joris Comhair, Giovanni Morelli, Jean-Michel Rigo, Rudi D'Hooge, Chadi Touma, Rupert Palme, Ilse Dewachter, Martin vandeVen, Robert J. Harvey, Serge N. Schiffmann, Elisabeth Piccart, Bert Brône

**Affiliations:** 1 Department of Neuroscience, UHasselt, 3500 Hasselt, Belgium; 2 Brain Development and Disease Laboratory, Instituto Italiano di Tecnologia, 16163 Genova, Italy; 3 Laboratory for Biological Psychology, University of Leuven, 3000 Leuven, Belgium; 4 Department of Behavioural Biology, University of Osnabrück, 49076 Osnabrück, Germany; 5 Institute of Biochemistry, University of Veterinary Medicine Vienna, Vienna A-1210, Austria; 6 School of Health, University of the Sunshine Coast, Sippy Downs, QLD, Australia; 7 Sunshine Coast Health Institute, Birtinya, QLD, Australia; 8 Laboratory of Neurophysiology, Université libre de Bruxelles, 1070 Brussels, Belgium

**Keywords:** Neuroscience, Behavioral neuroscience

## Abstract

The glycine receptor alpha 2 (GlyRα2) is a ligand-gated ion channel which upon activation induces a chloride conductance. Here, we investigated the role of GlyRα2 in dopamine-stimulated striatal cell activity and behavior. We show that depletion of GlyRα2 enhances dopamine-induced increases in the activity of putative dopamine D1 receptor-expressing striatal projection neurons, but does not alter midbrain dopamine neuron activity. We next show that the locomotor response to d-amphetamine is enhanced in GlyRα2 knockout animals, and that this increase correlates with c-fos expression in the dorsal striatum. 3-D modeling revealed an increase in the neuronal ensemble size in the striatum in response to D-amphetamine in GlyRα2 KO mice. Finally, we show enhanced appetitive conditioning in GlyRα2 KO animals that is likely due to increased motivation, but not changes in associative learning or hedonic response. Taken together, we show that GlyRα2 is an important regulator of dopamine-stimulated striatal activity and function.

## Highlights

• The dopamine-induced increase in SPN excitability is increased in GlyRα2 KO mice
• The locomotor response to d-amphetamine is increased in GlyRα2 KO mice
• Appetitive conditioning is enhanced in GlyRα2 KO mice

## Graphical abstract

**Figure:**
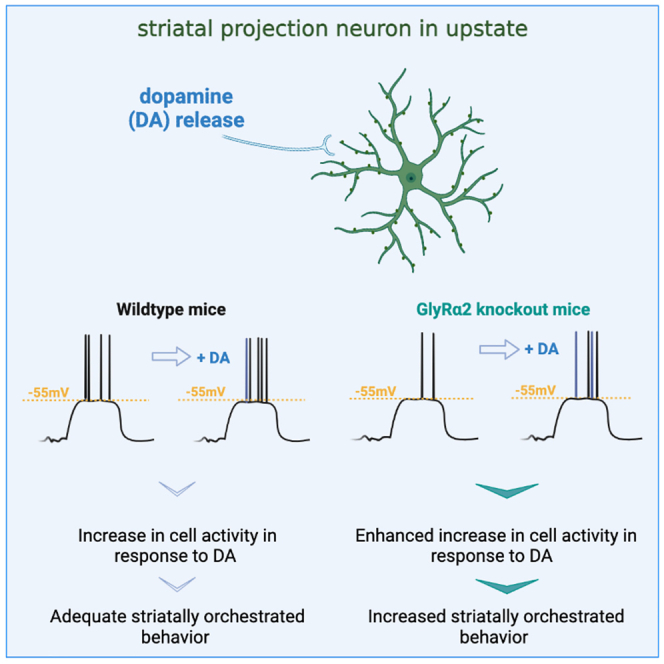

## Introduction

The dorsal striatum is the primary input hub to the basal ganglia and has a pivotal role in motor function. It is crucial to the execution of procedural memories as well as movement coordination and timing.^1^^,^^2^^,^^3^^,^^4^^,^^5^ The dorsal striatum is moreover critical for motivated behavior.^6^^,^^7^^,^^8^^,^^9^^,^^10^ Lesions to the dorsolateral striatum impair cue-motivated instrumental responding^11^ and viral restoration of dopamine signaling in dopamine-deficient mice in the nigrostriatal pathway rescues operant conditioning.^12^

Striatal projection neurons, which make up 95% of all striatal cells, receive dopaminergic inputs from the midbrain,^13^^,^^14^ and can be divided into cells that express the G_s_-coupled dopamine D1 receptor (DRD1) and cells that express the Gi-coupled dopamine D2 receptor (DRD2), although co-expression is reported as well.^15^^,^^16^^,^^17^ DRD1-expressing cells give rise to the direct pathway, projecting to the internal globus pallidus and substantia nigra pars reticulata. The DRD2-expressing SPNs project to the indirect pathway, relaying via the external globus pallidus and subthalamic nucleus. SPNs from both pathways cooperate to produce coherent goal-directed movements.^18^^,^^19^^,^^20^

At rest, all SPNs reside in a hyperpolarized resting state (around −80mV), i.e., the “downstate”, largely governed by inwardly rectifying potassium currents.^21^^,^^22^^,^^23^^,^^24^ In case of sufficient, converging glutamatergic innervation, which originates in the cortex or thalamus, SPNs transition to a near-threshold “upstate”. Inwardly rectifying potassium currents cease and L-type calcium channels start to open. When in upstate, dopamine promotes activation of direct pathway SPNs, while inhibiting indirect pathway SPNs: dopamine DRD1 activation increases excitability by negative modulation of K_v_1.2 channels, as well as small-conductance and big conductance potassium channels (SK and BK channels).^25^^,^^26^ DRD2 activation in upstate SPNs, however, decreases α-amino-3-hydroxy-5-methyl-4-isoxazolepropionic acid receptor (AMPAR) currents, mobilizes intracellular calcium, which causes a negative modulation of calcium Cav1.3 channels, increases potassium currents and decreases opening of voltage-gated sodium channels.^27^^,^^28^

In the present report, we investigated the potential of the glycine receptor alpha 2 (GlyRα2) to limit dopamine-induced increases in SPN excitability. Glycine receptors are ligand-gated ion channels that induce a fast increase in chloride conductance upon activation. They are either homo-pentamers, containing five alpha subunits, or a heteropentamers containing four alpha subunits and one beta subunit.^29^ There are four alpha subunits known: alpha 1–4. Until recently, the alpha 2 subunit was believed to be expressed throughout development, with expression declining toward adulthood in favor of alpha 1 and 3. We have demonstrated that GlyRα2 remains expressed in the adult dorsal striatum.^30^ Perforated patch voltage-clamp recordings showed that activation of GlyRα2 by 3mM glycine application induces a chloride current that drives the membrane potential toward the equilibrium potential of chloride (−54mV). When in downstate (i.e., −80mV), GlyRα2 activation thus depolarizes the cell. Indeed, application of GlyR antagonist strychnine reduced the holding current and both strychnine application and GlyRα2 KO hyperpolarize the cell membrane potential. However, when the membrane potential depolarizes to exceed the equilibrium potential of chloride, GlyRα2 activation causes an inhibitory chloride current and shunts the depolarization.^30^ Shunting inhibition by GlyRα2s is thus expected to be most significant when dopamine inputs enhance glutamatergic inputs in an upstate D1-expressing SPN. Importantly, GlyRα2 is the only functionally expressed glycine receptor in the dorsal striatum, which is critical for motor behavior, habit formation and motivated behavior.^8^^,^^11^^,^^12^^,^^31^^,^^32^ Indeed, 3mM glycine application induces pronounced chloride currents in SPNs from mouse brain slices from wildtype mice, but elicits no response in SPNs from their GlyRα2 knockout littermates.^30^ These GlyRs are thus ideally positioned to alter striatal function. We hypothesized that depletion of GlyRα2 further increases dopamine-boosted activity in upstate SPNs, and consequently, enhance basal-ganglia-orchestrated behavior.

## Results

### Lack of GlyRα2 enhances dopaminergic modulation of striatal excitability

**Figure 1:**
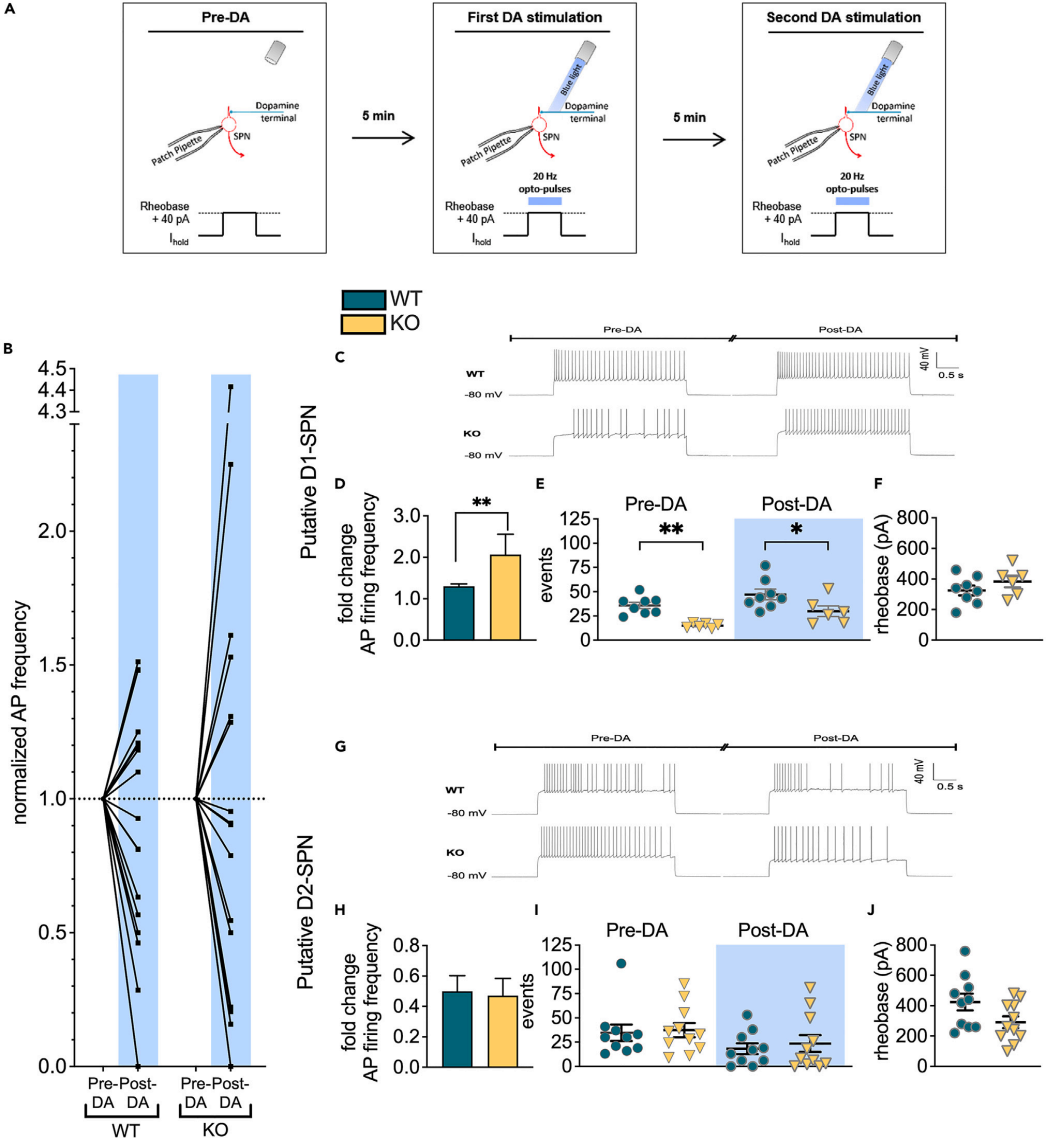
Lack of GlyRα2 enhances dopaminergic modulation of striatal excitability (A) Graphical protocol of dopaminergic activity modulation in SPNs. (B) Dopamine release in the striatum alters the relative intrinsic activity, measured as action potential (AP) frequency divided by baseline AP frequency, in WT (n = 18) and GlyRα2 KO (n = 17) mice. In both WT and GlyRα2 KO SPNs, a subpopulation of cells increased firing activity, whereas another subpopulation decreased firing activity. In agreement with distinct dopaminergic modulation of D1-versus D2-SPNs, we termed the subpopulations pD1-or D2-SPNs (pD1-SPN, pD2-SPN respectively), and analysis was conducted separately. (C) Representative traces of evoked action potentials recorded before and after dopamine modulation in pD1-SPNs of WT and GlyRα2 KO mice. (D) Dopamine-induced activity increase in pD1-SPNs is enhanced in GlyRα2 KO mice, expressed as fold change of action potential frequency after the second optogenetic stimulation of dopamine neurons compared to before optogenetic stimulation (n_WT_ = 8; n_KO_ = 6). (E) GlyRα2 KO mice exhibit a decrease in the number of action potentials fired in pD1-SPNs, both before and after optogenetic stimulation (n_WT_ = 8; n_KO_ = 6). (F) The rheobase was not significantly different between WT and GlyRα2 KO animals in pD1-SPNs. (G) Representative traces of evoked action potentials recorded before and after dopamine modulation in pD2-SPNs of WT and GlyRα2 KO mice. (H) Dopamine-induced activity decrease in pD2-SPNs is unaltered in GlyRα2 KO mice compared to WT littermates (n_WT_ = 10; n_KO_ = 11). (I) The number of action potentials fired in pD2-SPNs is similar in WT and GlyRα2 KO mice, before and after optogenetic stimulation (n_WT_ = 10; n_KO_ = 11). (J) The rheobase was not significantly different between WT and GlyRα2 KO animals in pD2-SPNs. See also Figure S1. Data are represented as mean ± SEM. ∗p < 0.05, ∗∗p < 0.01.

Dopamine release to upstate D1-SPNs enhances cell activity, whereas GlyRα2 activation causes shunting inhibition when the cell becomes depolarized above the equilibrium potential of chloride (i.e., −54mV).^30^ We therefore hypothesized that depletion of GlyRα2 would allow a larger increase in SPN activity in response to dopamine. To evaluate our hypothesis, we performed patch clamp electrophysiology recordings on acutely isolated brain slices from GlyRα2 KO or WT animals. In order to investigate our hypothesis, we first injected current (rheobase +40pA) and measured SPN excitability (“pre-DA”). Five minutes later, we optogenetically induced dopamine release to SPNs. Another 5 min later, we optogenetically stimulated again and measured SPN excitability (“post-DA”) in both WT and GlyRα2 KO (Figure 1A). The protocol was designed to ensure onset of dopaminergic modulation and limit variability: there is a sustained dopaminergic modulation of D1-SPN excitability up to 10 min with the least variability between 5 and 6 min (Figure S1).^25^ Further, in whole-cell configuration there is a delayed response compared to perforated-patch with a variable onset of the modulation. This will make the interpretation of dopamine-modulated activity during the first pulse of 10 s variable and inconclusive.

We found two SPN subpopulations: one that was excited and one that was inhibited after dopamine modulation (Figure 1B). Since DRD1-expressing SPNs in upstate enhance activity in response to DA, and DRD2-expressing SPNs in upstate decrease activity in response to DA, (18, 19, 24, 25) we termed these cells putative D1 or D2 SPNs (pD1-SPNs and pD2-SPNs respectively) and performed further analysis on these separate populations. GlyRα2 deletion enhanced dopamine modulation of the activity of pD1-SPNs. Optogenetic stimulation increased the action potential (AP) frequency by a factor of 2.067 in GlyRα2 KO, while in WT an increase of 1.302 was observed (Figures 1C and 1D). However, dopamine-modulated activity of pD2-SPNs was not altered in GlyRα2 KO compared to WT (Figures 1G and 1H). Counterintuitively, we found that somatic current injection induced less AP firing in pD1-SPNs of GlyRα2 KO compared to WT animals. However, this difference in AP firing between genotypes became smaller after dopamine modulation, which explains the relative increase of firing pre- and post-dopamine modulation in GlyRα2 KO animals (Figure 1E). In pD2-SPNs, AP firing did not differ significantly between GlyRα2 KO and WT (Figure 1I). GlyRα2 KO showed no differences in rheobase (Figures 1F and 1J) or membrane resistance (Table S2) of pD1-or pD2-SPNs when compared to WT controls. To verify that the current injection by itself would not affect intrinsic firing, we included SPN recordings of optogenetic-inducible WT with a similar current injection protocol, but without the optogenetic stimulation. We did not observe any significant deviations in firing rates at the beginning and end of the protocol (Figure S2D). Taken together, we show enhanced dopaminergic modulation of pD1-SPN activity in GlyRα2 KO, and reduced intrinsic excitability.

### Depletion of GlyRα2 does not affect dopamine neuron activity

**Figure 2:**
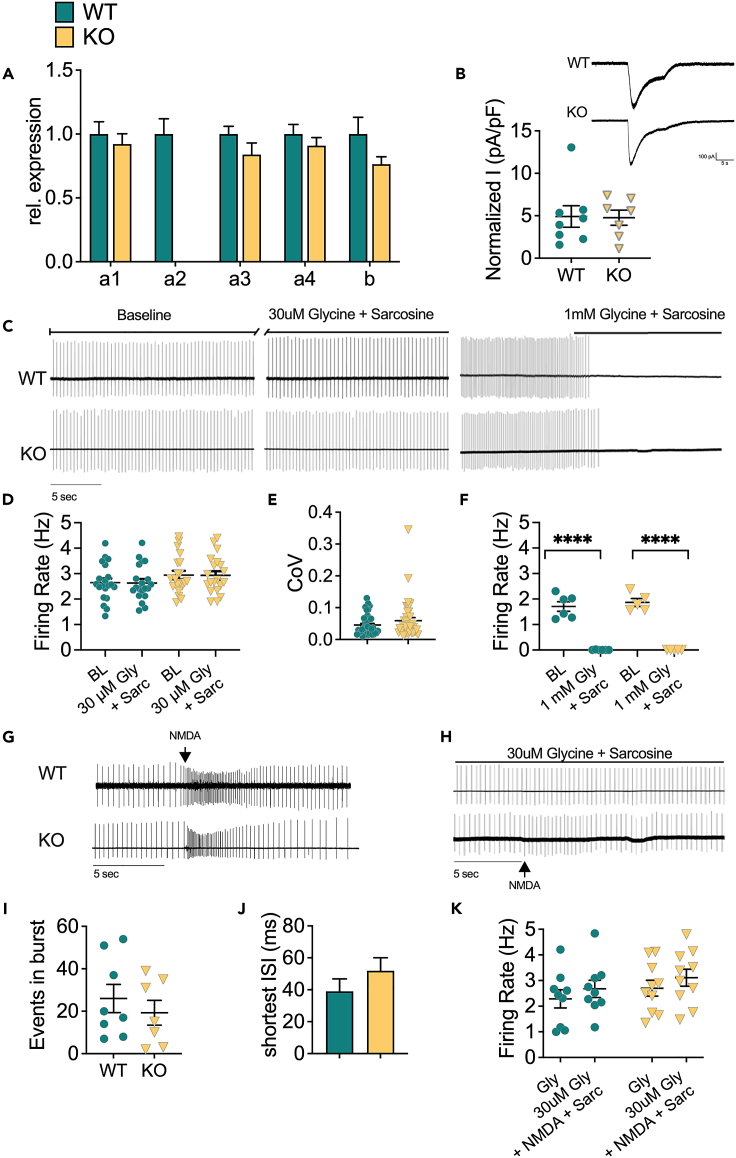
Depletion of GlyRα2 does not affect dopamine neuron activity (A) Relative expression of GlyR subunits is unaltered between GlyRα2 KO and WT littermates, with the exception of α2. (B) Normalized current density is unaltered in GlyRα2 KO animals (inset: exemplar traces of glycine-induced currents in dopamine neurons from WT (top) and GlyRα2 KO (bottom) animals). (C) Exemplar traces of pacemaking firing without glycine application (left), with 30 μM glycine and sarcosine (500 μM) application (middle), and with 1 mM glycine and sarcosine (500 μM) application. (D) The baseline firing rate is unaltered in GlyRα2 KO animals, compared to WT littermates. Moreover, there is no change in baseline firing rate in response to 30 μM glycine and sarcosine (500 μM) in GlyRα2 KO nor WT mice. (E) Dopamine cells from GlyRα2 and WT littermates fire equally regularly. (F) Glycine (1 mM) and sarcosine (500 μM) completely abolishes cell firing in both GlyRα2 KO and WT littermates. (G) Exemplary traces of burst firing induced by NMDA iontophoresis in WT (top) and GlyRα2 KO animals (bottom). (H) Exemplar traces of the response to NMDA iontophoresis when 30 μM and sarcosine (500 μM) is co-applied in WT (top) and GlyRα2 KO mice (bottom). (I) The number of events in a burst is unaltered in GlyRα2 KO animals compared to WT littermates. (J) The shortest inter-spike interval in a burst is unaltered in GlyRα2 KO animals compared to WT littermates. (K) NMDA iontophoresis is unable to evoke burst firing in both GlyRα2 KO and WT animals in the presence of 30 μM glycine and sarcosine (500 μM) application. See also Figure S2. Data are represented as mean ± SEM. ∗∗∗∗p < 0.0001.

A switch occurs from homomeric GlyRα2 in neonatal to heteromeric α1/β in adult dopamine neurons of the substantia nigra, similar to what has been described for the spinal cord and brainstem.^33^ It is not clear whether remaining GlyRα2 signaling at adult age alters dopaminergic activity. Therefore, we first determined the GlyR subunit expression in the substania nigra pars compacta (SNc) profile using real-time polymerase chain reaction (RT-PCR) (Figure 2A). As expected, GlyRα2 KO exhibit a complete loss of GlyR α2 subunit mRNA, with no changes in the expression of other GlyR subunit genes.

We next sought to determine the role of GlyRα2 in SNc dopamine neuron pacemaking firing. First, with fast-application of glycine (1 mM) we were able to evoke currents in WT as well as GlyRα2 KO, without any difference (Figure 2B). Next, we measured pacemaking firing in SNc DA neurons in the presence of GABA_A_ receptor blockers, as GABA signaling can affect DA neuron firing. GlyRα2 KO showed no differences in baseline pacemaking activity compared to WT (Figures 2C and 2D), and dopamine neurons fired with a similar inter-spike interval (Figures S2A and S2B) and fired equally regularly (Figure 2E), also during bursts (Figure S2C). Indeed, a tonic current did not appear to cause differences in pacemaking firing, as we found no change in firing rate upon strychnine application (Figures S2D and S2E). To exclude indirect effects mediated by GABA_A_ receptors, we also measured the firing rate in the absence of GABA_A_R antagonists, but no difference was apparent in firing rate between cells from WT and GlyRα2 KO (Figures S2F and S2G). In order to investigate influences of GlyR activation on pacemaking activity, we bath-applied glycine both at a low concentration, which is thought to activate high affinity extrasynaptic GlyRs, and at a high concentration, which activates low-affinity synaptic receptors. Application of low levels of glycine (30 μM) did not alter firing rates (Figures 2C and 2D). However, high glycine concentrations (1 mM) inhibited pacemaking activity completely in both GlyRα2 KO and WT controls (Figures 2C and 2F). These findings confirm the presence of functional GlyRs in DA cells of both genotypes, and suggest little to no contribution of GlyRα2 at these high glycine concentrations. Since tonic inhibitory currents mediated by GABA_A_ receptors can suppress burst firing,^34^ it could be expected that GlyR-mediated tonic currents result in similar effects. We therefore measured burst activity induced by N-methyl-D-aspartate (NMDA) (50 mM) iontophoresis in GlyRα2 KO and WT (Figure 2G). Burst activity measured by loose cell patch clamp showed no differences in number of events per burst (Figure 2I), shortest inter-spike interval (Figure 2J), mean inter-spike interval (Figure S2B) or burst regularity (Figure S2C). In contrast to pacemaking activity, application of low glycine concentrations (30 μM) completely blocked burst firing in both GlyRα2 KO and WT mice (Figures 2H and 2K). These data confirm a role for GlyRs in activity modulation of dopamine neurons, but independent of the α2 subunit.

### *In vivo* increased dopamine neurotransmission enhances striatal activation and locomotor behavior in GlyRα2 KO mice

**Figure 3:**
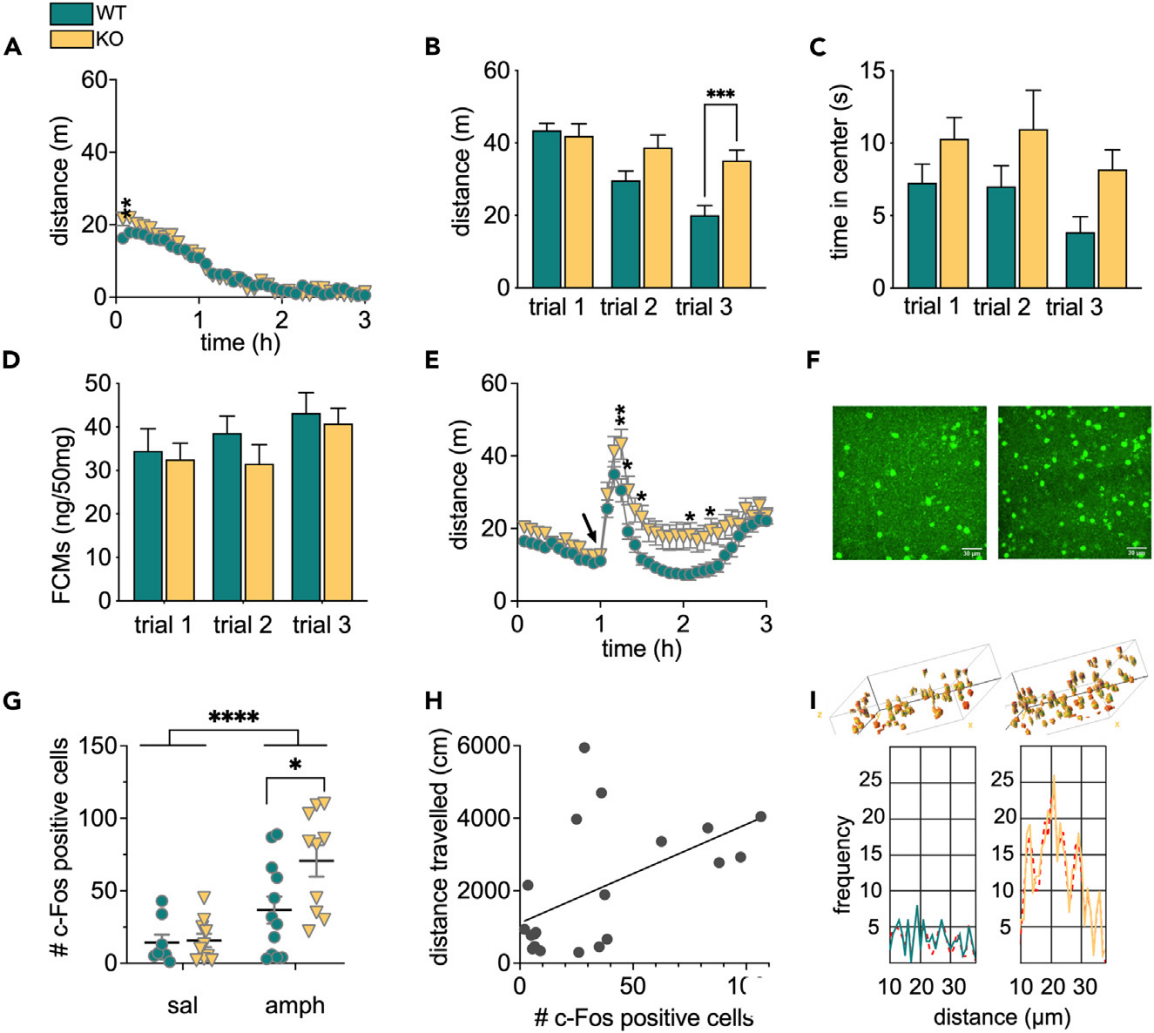
*In vivo* increased dopamine neurotransmission enhances striatal activation and locomotor behavior in GlyRα2 KO mice (A) GlyRα2 KO animals exhibit enhanced locomotor responding in a novel environment that disappears over time. (B) Consecutive open field assessments (1-week intertrial interval) reveal increased distance run in GlyRα2 KO animals compared to WT littermates in the third trial. (C) GlyRα2 KO animals spend more time overall in the center of the arena, compared to WT animals. (D) Levels of fecal corticosterone metabolites (FCMs) remain unaltered in GlyRα2 KO animals over consecutive trials compared to WT littermates. (E) GlyRα2 KO animals exhibit an increased locomotor response to 5 mg/kg D-amphetamine (i.p.). (F) Representative images of c-fos immunohistochemistry (IHC) after amphetamine (5 mg/kg, i.p.) administration in WT (left) and GlyRα2 KO (right). Scale bar represents 30 μm (G). The number of c-fos positive cells increased in amphetamine-treated GlyRα2 KO animals, compared to WT littermates. (H) Distance traveled positively correlates to the number of c-fos positive cells. (I) Histograms plotting the frequency that a distance between two cells was measured and indicate a similar distribution of c-fos-positive cells in WT and KO mice, suggesting similar numbers of cells within cell strings (full lines: raw data, dotted red lines: Gaussian fits). See also Table S3. Insets are examples of 3D-modeling of c-fos-positive cells. Data are represented as mean ± SEM. ∗p < 0.05; ∗∗p < 0.01; ∗∗∗∗p < 0.0001.

Behavioral output is mediated by a group of causally related co-active neurons, known as a neuronal ensemble, and dopamine inputs to the striatum increase the size of the neuronal ensembles.^35^ We wanted to assess whether the enhanced response to dopamine in pD1-SPNs in GlyRα2KO animals affects locomotion and neuronal ensemble size. We first recorded locomotor activity in WT and GlyRα2 KO after either saline to evaluate baseline differences in activity (Figure 3A) or D-amphetamine (5 mg/kg, Figure 3E) administration. In the saline control experiment, GlyRα2 KO exhibited an enhanced activity when first placed in the novel environment compared to WT, but this difference vanished over time (Figure 3A). To further explore potential differences in open field anxiety, we performed a repeated open field experiment and measured fecal corticosterone metabolites (FCMs).^36^ GlyRα2 KO show an overall increased total distance run, distance run decreases over trials in both KO and WT, and an interaction between genotype and trial is also present (Figure 3B). We observed that GlyRα2 KO spent overall more time in the center of the arena over trials (Figure 3C). We measured similar levels of corticosterone metabolites in WT and KO (Figure 3D).

We furthermore report that treatment with D-amphetamine (5 mg/kg) caused an excessive locomotor response in GlyRα2 KO compared to WT (Figure 3B), which was absent in response to cocaine (20 mg/kg) (Figure S3). To correlate the behavioral response to excessive striatal activation, we quantified the expression of immediate-early gene (IEG) c-fos after acute saline or D-amphetamine treatment in GlyRα2 KO and WT (Figures 3F–3H), often used as a tool to study neuronal ensembles that are activated by drug self-administration.^37^^,^^38^^,^^39^^,^^40^ In addition, c-fos expression is particularly useful within the framework of integration of dopaminergic and glutamatergic inputs to the striatum: while glutamatergic inputs to the striatum can induce c-fos expression, dopamine and glutamate input to SPNs in upstate combined significantly enhance c-fos expression.^41^^,^^42^^,^^43^ In agreement with an excessive response to dopaminergic input in SPNs, we detected an increase in the number of c-fos positive cells after D-amphetamine treatment 2 h after injection, when c-fos protein expression typically peaks.^44^ In response to saline administration, both genotypes showed a similar number of activated cells, but in response to amphetamine, this is higher in GlyRα2 KO than WT animals (Figure 3G). We directly correlated distance traveled by a subject with the number of c-fos positive cells for that subject, and found a significant correlation, confirming the behavioral relevance of c-fos staining (Figure 3H). Dopamine release to the striatum can activate neuronal ensembles, and increased synchronous dopamine release can increase ensemble size. Small step angular variation of the images indeed revealed a regional alignment of the c-fos-positive cells in neuronal ensembles after amphetamine stimulation in the striatum, indicating a mechanism of co-activation at times of high dopamine release. Concurrently, histograms plotting the frequency that a distance between two cells was measured (Figure 3I) confirm an increase in number of c-fos positive cells in GlyRα2 KO, yet, the distribution (i.e., at which distances are the peaks located) are comparable between WT and KO, with the highest amplitudes situated around 12, 20 and 28 μm (raw data: Table S3).

### Depletion of GlyRα2 increases reward-motivated behavior

**Figure 4:**
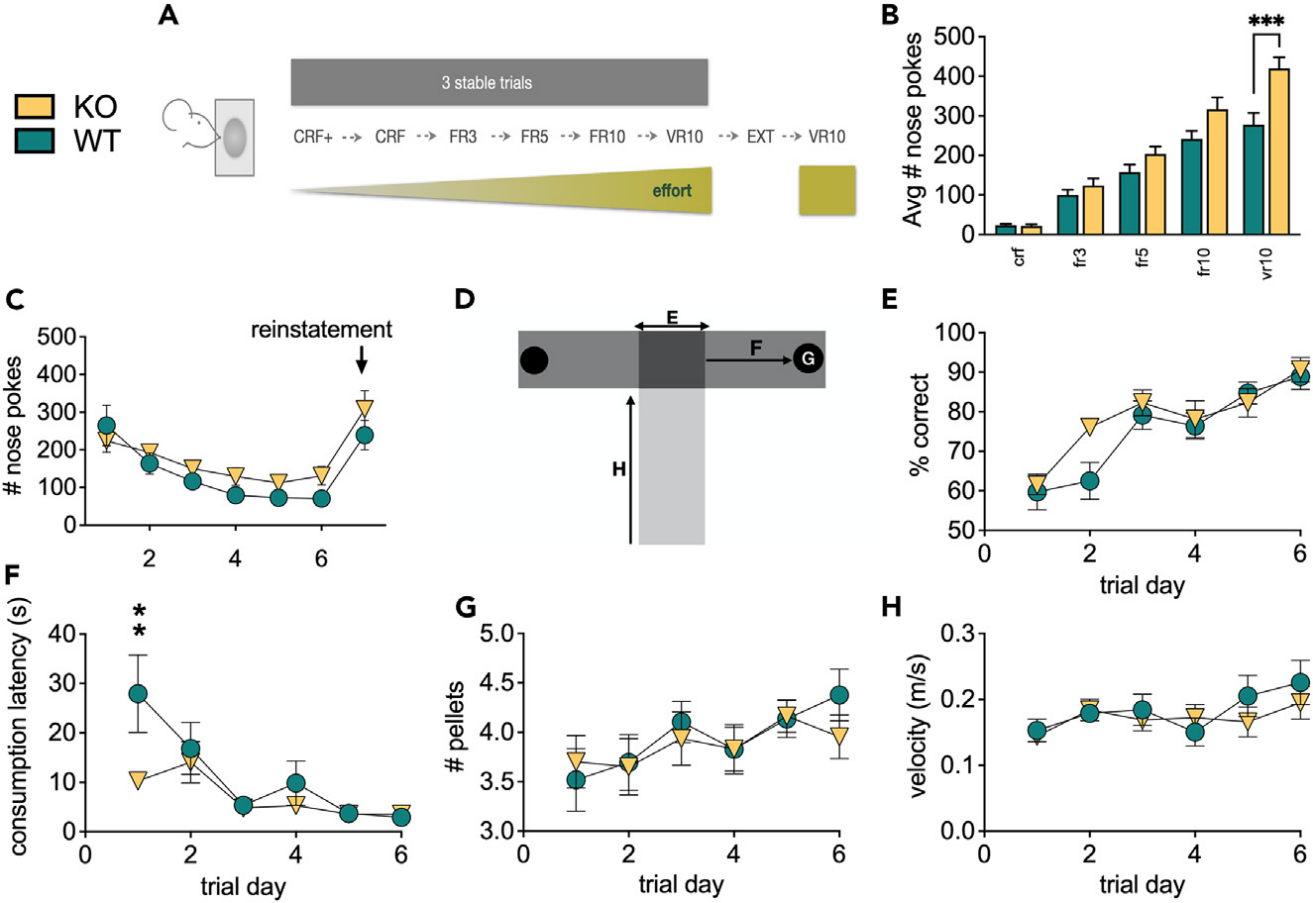
Depletion of GlyRα2 increases reward-motivated behavior (A) Graphical representation of appetitive conditioning with increasingly more demanding reward schedules. (B) GlyRα2 KO animals exhibit enhanced responses on a vr10 reward schedule (summary bars are the average of the three days of stable nose poking within the given reward schedule; crf+: continuous reinforcement + reward every 120 s; crf: continuous reinforcement; fr3: fixed ratio 3; fr5: fixed ratio 5; fr10: fixed ratio 10; vr10: variable ratio 10). (C) GlyRα2 KO animals exhibit faster extinction rates and similar reinstatement (indicated by the arrow), compared to WT. (D) Scheme of the T-maze, indicating the data plotted in graph E–H. (E) GlyRα2 KO animals show unaltered % correct arm entries, indicative of unchanged associative learning. (F) GlyRα2 KO show a decreased time to start consuming the reward once a correct arm entry was made compared to WT, indicative of enhanced motivation. (G) GlyRα2 KO and WT littermates exhibit a similar number of pellets consumed, indicative of unaltered hedonic response. (H) GlyRα2 KO and WT littermates approach the decision box (dark gray area in Fig. D) with the same velocity. Data are represented as mean ± SEM. ∗∗p < 0.01, ∗∗∗p < 0.005.

The dorsal striatum is crucial to motivated behavior. We therefore hypothesized that depletion of GlyRα2 would increase motivated behavior. To test this, we performed an appetitive conditioning task, in which animals were trained on increasingly demanding reward schedules (acquisition), followed by an extinction and reinstatement phase (Figure 4A) in GlyRα2 KO and WT. In the acquisition phase, stable measurements were required over three consecutive days before mice proceeded to the next schedule, as described by Piccart et al.^45^ to ensure similar training levels between both groups, avoiding overtraining in one group compared to the other. The mean values for each reward schedule (i.e., average of performance on three stable days) were plotted (Figure 4B). A *post hoc* test showed significantly increased performance during the most demanding task in GlyRα2 KO compared to WT. In the subsequent extinction phase, GlyRα2 depletion caused a dramatic drop in number of nose pokes relative to the last conditioning trial (Figure 4C). Reinstatement occurred for both genotypes in a similar manner.

Enhanced appetitive conditioning can be the result of enhanced associative learning, hedonic response, or motivation. In order to pick apart the components that might be affected by GlyRα2 depletion we performed a T-maze, as described by Robinson et al., 2005^46^ (Figure 4D). We did not observe a main genotype effect on correct arm entries, indicating no differences in associational learning (Figure 4E). We observed an overall enhanced motivation, evident in a decreased latency to consumption once the correct path arm was chosen. However, this difference disappeared over trials as animals hit a floor effect (Figure 4F). Animals consumed more pellets over trial days, an effect that was similar in WT and GlyRα2 KO (Figure 4G), indicative of unaltered hedonic response. Finally, WT and KO did not differ in run velocity, which similarly increased over trials (Figure 4H).

## Discussion

The dorsal striatum is a coordinating hub that provides the main input to the basal ganglia. Converging glutamatergic input bring SPNs to a near-threshold upstate. Concurrent dopamine release will further enhance SPN excitability. The present work investigated the potential of the GlyRα2 to affect dopaminergic modulation of SPNs in upstate, overall striatal activation, as well as striatally orchestrated behavior.

To investigate whether DA release to the dorsal SPNs increases striatal cell activity, we optogenetically stimulated DA terminals and recorded action potentials from SPNs in upstate. Optogenetic stimulation appropriately mimics synchronized phasic DA release, evoking DA concentrations in the sub-micromolar range, similar to *in vivo* DA transients.^25^^,^^47^ Dopamine input to DRD1-expressing cells in upstate enhances SPN cell excitability, whereas dopamine input to upstate DRD2-expressing cells decreases cell excitability.^25^ Indeed, we report that optogenetic DA release to SPNs in upstate either increases or decreases action potential frequency in both WT and GlyRα2 KO animals. We therefore termed these cells either putative D1- (pD1) or putative D2- (pD2) SPNs. It is noteworthy that Prager et al. (2020) found that DA modulates D1-SPNs in striatal striosomes and matrix in an opposite manner: DA uncaging onto striosome D1-SPNs decreased upstate duration, whereas DA uncaging onto matrix D1-SPN increased upstate duration, and this modulation depended on L-type voltage-gated calcium channels. While we cannot exclude that a fraction of the pD2-SPNs are in fact striosomal D1-SPNs, it seems unlikely given that L-VGCCs do not affect SPN excitability. In addition, the effects of dopamine on upstate duration followed a U-shape, and thus crucially depend on experimental design, and DA-induced shortening of striosomal D1-SPN upstate duration was very modest compared to the increase seen in matrix D1-SPNs.^25^^,^^48^

The DA-induced increase in action potential frequency in pD1-SPNs was however significantly more pronounced in GlyRα2 knockout animals. Striatal GlyRα2 are thought to be extrasynaptic receptors that produce a tonic current.^30^ At low transmembrane conductances, shunting inhibition may not provide strong inhibitory effects on the cell. However, high GlyRα2 conductance can be inhibitory through shunting, thereby decreasing firing probability of the cell, similar to the inhibitory effects of high tonic GABA_A_R conductances.^49^

We next demonstrated that *in vivo* administration of amphetamine increases the size of the neuronal ensemble that is activated, evidenced by increased c-fos- positive cells, which correlated to the increase in locomotor responses. Indeed, the dorsal striatum is critically involved in the motor response to psychostimulants. Dopamine efflux significantly increases in response to d-amphetamine administration.^50^ Ablation of DRD1-expressing SPNs in the dorsomedial striatum causes a reduction in the locomotor response to d-amphetamine.^51^ Administration of cocaine causes a sharp rise in intracellular calcium levels in DRD1-expressing neurons in the dorsal striatum. Notably, neuronal ensembles revealed 3-D modeling of confocal c-fos imaging revealed strings of activated cells, in agreement with sparse active zone-like dopamine release sites.^52^ The number of cells within these strings did not differ between WT and KO animals. This suggests that the amount of dopamine released within a sparse active zone-like release site is similar in WT and KO animals.

We note that in spite of the increase in activated neuronal ensemble size in response to d-amphetamine, a larger relative increase in action potential firing frequency after optogenetic dopamine release and enhanced forward locomotor response, the absolute number of action potentials remained lower in GlyRα2 KO animals. In agreement with the present results did we report decreased firing rate in SPNs of GlyRα2 KO animals in Molchanova et al., 2017.^30^ We speculate that the decreased discharge might be due to the expression of voltage-gated calcium (Cav1) channels in the dendrites of SPNs where most cortical, glutamatergic input arrives. When in upstate, D1 receptor activation enhances the calcium currents mediated by Cav1 through a DARPP-32 signaling cascade. In GlyRα2 KO mice, the lack of glycinergic shunting inhibition, can enhance activation of voltage-gated calcium channels, and thereby consequently also enhance their inactivation, i.e., their transition into a nonconducting state. Indeed, it was earlier shown that GlyRα2 activation in the neonatal brain, where GlyRα2 activation is depolarizing, similar to an adult SPN in upstate, activates voltage-gated calcium channels and promotes calcium influx.^53^ In addition, voltage-gated sodium channels might also enter a non-conductive state, further adding to the decreased excitability in GlyRα2 KO mice. With regards to the apparent discrepancy with c-fos expression, we must draw a distinction between activity at the population level (i.e., the neuronal ensembles) and at the level of a single cell. C-fos activity is used to study neuronal ensembles that encode associations between drug-related cues and psychostimulants (for a review, see^54^), and the threshold for c-fos expression is lower than for action potential firing. Enhanced cell activation, evidenced by increased size of the neuronal cell ensemble, enhanced sensitivity to dopaminergic modulation, but decreased single cell firing frequency suggest that neuronal ensemble size and the change in firing frequency, rather than the absolute frequency, dictates the behavioral response. Indeed, Maltese et al. (2021) revealed that low doses of DRD1/2 receptor agonists did not alter the number of D2-SPNs that were recruited during forward locomotion, but often increased D1-SPN ensemble sizes.^35^ Similarly, reward delivery increased the size of the direct pathway SPN ensemble. Moreover, calcium transient properties in individual cells did not change.

Surprisingly, depletion of GlyRα2 enhanced the locomotor response to amphetamine, but not cocaine. This may be due to the distinct pharmacological profile of cocaine and amphetamine,^55^ with cocaine showing a much lower potency to induce locomotor behavior compared to amphetamine.^56^ Indeed, the locomotor response to amphetamine, but not cocaine, exhibits a biphasic pattern that is typical for an enhanced response.^57^ Moreover, high amphetamine concentrations inhibit the degradation of dopamine, while activating its synthesis, augmenting the vesicular release, and enhances phasic dopamine signaling.^58^^,^^59^^,^^60^ The differential effect of GlyRα2 depletion on baseline locomotion and amphetamine-induced locomotion might be explained by the specific role of GlyRα2 in phasic DA release. We found that pD1-SPNs of GlyRα2KO mice exerted an enhanced dopamine-modulated activity at times of phasic dopamine release. This could explain the absence of differences at baseline locomotor behavior and corresponding c-Fos expression in GlyRα2KO mice and littermates. During basal locomotion, there is little phasic dopamine signaling within the striatum. These findings are in accordance with a previous study of Molchanova et al. (2017), which showed no alterations in basal locomotor activity of GlyRα2KO.^30^ However, GlyRα2KO mice showed impairments during motor learning tests, during which proper phasic dopaminergic signaling is essential.^30^

We speculate that the observed increase in novelty-induced locomotion in the open field of GlyRα2KO mice is caused by these phasic dopamine responses as well. It is extensively reported that novel environments induce phasic dopaminergic activity in both animal models and humans.^61^^,^^62^^,^^63^ Additionally, we explored whether lack of GlyRα2 affects an acute stress response. Acute stress enhances the activity of the central amygdala.^64^^,^^65^ Enhanced activity within the central amygdala in turn dramatically increases locomotor as well as incentive, motivated behavior.^66^^,^^67^^,^^68^ At rest, GABA inputs to the amygdala inhibit its activity, and stress-induced hyperactivity of the amygdala always coincides with the removal of inhibition.^69^ Although the inhibitory control is predominantly controlled by GABAergic input, the central amygdala also expresses GlyRα2β and GlyRα3β heteromers with a minor component of GlyRα2 and GlyRα3 homomers.^66^^,^^67^^,^^70^^,^^71^^,^^72^ Nonetheless, GlyRα2 showed increased time spent in the center of the arena and no changes in corticosterone metabolites, rending an increased stress response unlikely.

In the aforementioned experiments, we controlled dopamine release onto SPNs. However, midbrain DA neurons also express glycine receptors, and we investigated whether lack of GlyRα2 alter dopamine neuron activity as well. Dopamine neurons fired at the same rate in WT and GlyRα2 KO, and inter-spike intervals were equally regular. Low concentration glycine perfusion did not affect firing rate in either WT or GlyRα2 KO, suggesting that low affinity, extrasynaptic glycine receptors do not control basal firing. However, high concentration glycine fully inhibited pacemaking firing in both WT and GlyRα2 KO mice. It could be surmised that the expression of GlyRα1 and GlyRα3 compensate for the loss of GlyRα2, or that GlyRα2, even when present, has little effect on SNc neuronal activity. In agreement with compensation by other GlyR subtypes, we found that normalized current amplitudes in response to glycine application were similar in WT and GlyRα2 KO. We cannot exclude, however, that in spite of similar firing patterns in GlyRα2 KO and WT animals, there may be differences in dopamine release due to altered vesicle filling or release probability. Yet, as aforementioned, based on the findings by Liu et al. (2018), increased vesicular release (probability) would likely reveal itself in a shift in inter-cell distance histograms (i.e., an increase in cells within a string of activated cells, likely activated by DA released from one sparse active zone-like DA release site), which was not the case.^52^ Taken together, we conclude that depletion of GlyRα2 most significantly modulates basal ganglia signaling at the level of the striatum.

While the dorsal striatum is typically linked to its role in motor behavior, it is now clear that the dorsal striatum plays a crucial role in reward-motivated behavior as well. Input from the substantia nigra to the dorsal striatum is also critical for motivated behavior.^6^^,^^7^^,^^8^^,^^9^^,^^10^ Viral restoration of dopamine signaling in the nigrostriatal pathway rescues operant conditioning,^12^ lesions to the dorsolateral striatum impair cue-motivated instrumental responding,^11^ motivated attraction to an incentive stimulus is strengthened upon injections with indirect dopamine agonist amphetamine into the dorsolateral striatum,^31^ and inhibiting neuronal activity in the dorsal striatum by microinjections of baclofen/muscimol decreases cocaine self-administration.^32^ In humans, strong activation using fMRI is reported in the dorsal striatum in response to a reward-conditioned stimulus.^73^ Since we revealed enhanced responses to dopaminergic input in the dorsal striatum, we hypothesized excessive reward-motivated behavior in GlyRα2 KO. We report that depletion of GlyRα2 causes excessive performance in an appetitive conditioning task. This is in agreement with the reported increase in ethanol consumption in mice lacking GlyRα2^74^ or mice that express ethanol-insensitive GlyRα2 subunits.^75^ In our appetitive conditioning task, behavioral differences between GlyRα2 KO and WT controls only became apparent during highly demanding motivational reward schedules, indicative of enhanced motivated behavior. Accordingly, striatal dopamine depletion-induced impairment in appetitive conditioning only becomes apparent on highly demanding reward schedules,^76^ and increasing striatal activation by inhibition or depletion of phosphodiesterase 10A hinders appetitive conditioning only at highly demanding reward schedules.^45^^,^^77^ T-maze performance confirmed excessive motivated behavior. Our data further suggest that the hedonic response is unaltered in KO animals. We speculate that this may be because these are mediated by hedonic hotspots in the ventral striatum where GlyRα1 and GlyRα3 are also present.^78^^,^^79^^,^^80^^,^^81^ During extinction trials, KO animals immediately perform at a level that is comparable to their WT littermates. However, given the significantly increased performance on the last reward schedule of appetitive conditioning, the decrease in performance from rewarded to extinction trials is larger in KO animals. This is in agreement with reports that show that increasing activity of the dorsal striatum by intra-striatal injection of a partial NMDAR agonist enhances extinction of appetitive conditioning.^82^ Similarly, inactivation of the dorsolateral striatum by intra-striatal injection of sodium channel blocker bupivacaine impaired extinction.^83^ Finally, it seems unlikely that our results were mediated by altered stress responses in KO, as they showed similar levels of corticosterone metabolites in a repeated open-field task and spent a larger amount of time in the center of the arena.

Taken together, we show that depletion of GlyRα2 enhances dopaminergic modulation of striatal excitability and increases the size of activated cell ensembles. At the behavioral level, we report an increased locmotor response to d-amphetamine as well as increased appetitive conditioning.

### Limitations of the study

We note that GlyRα2 is not exclusively expressed within the dorsal striatum, and our experiments do not allow us to causally link changes in dorsal striatum cell activation to altered behavior. For instance, GlyRα2 is expressed within the ventral striatum, where it was shown to alter ethanol intake. Moreover, as discussed earlier, GlyRα2 is expressed within the amygdala, and we cannot exclude effects on the behavior measured, in spite of the lack of corticosterone metabolite changes. Moreover, GlyRα2s regulate migration and maturation of cortical neurons, and depletion of GlyRα2 alters glutamatergic circuitry and synaptic plasticity in the cerebral cortex.^53^^,^^84^ Within the striatum, GlyRα2 promotes the functional maturation of glutamatergic synapses on MSNs. We circumvent these developmental changes to a certain degree by mimicking an upstate using current clamp, rather than stimulating cortical inputs. However, at the behavioral level, they are likely to contribute to changes in GlyRα2 knockout animals.

## STAR★Methods

### Key resources table

**Table:** 

REAGENT or RESOURCE	SOURCE	IDENTIFIER
**Antibodies**		

c-Fos Antibody (2H2)	Novus Biologicals	NBP2-50037; RRID:AB_2665387

**Chemicals, peptides, and recombinant proteins**		

CNQX	Tocris Bioscience	Cat. No. 0190
CGP 54626 hydrochloride	Tocris Bioscience	Cat. No. 1088
DhβE	Tocris Bioscience	Cat. No. 2349
D-serine	Sigma-Aldrich	S4250
SR 95531 hydrobromide (Gabazine)	Tocris Bioscience	Cat. No. 1262
Glycine	VWR	101196X
Kynurenic acid	Sigma-Aldrich	K3375
L-689,560	Tocris Bioscience	Cat. No. 0742
NMDA	Sigma-Aldrich	M3262
Picrotoxin	Tocris Bioscience	Cat. No. 1128
Sarcosine	Sigma-Aldrich	131776
Strychnine	Sigma-Aldrich	S0532
Amphetamine	Tocris Bioscience	Cat. No. 2813
Cocaine hydrochloride	Fagron	Cat. No. 0244517
DAPI	Thermo Fisher Scientific	D1306
Fluoromount-G Mounting Medium	Thermo Fisher Scientific	00-4958-02
QIAzol Lysis Reagent	Qiagen	Cat. No. / ID: 79306
RNeasy Mini Kit	Qiagen	Cat. No. / ID: 74104
High-Capacity cDNA Reverse Transcription Kit	Applied Biosystems	4368814
Fast SYBR Green Master Mix	Applied Biosystems	4385612

**Experimental models: Organisms/strains**		

Mouse: C57BL/6J; GlyRα2^Y/+^	Robert J. Harvey^54^	N/A
Mouse: C57BL/6J; GlyRα2^Y/-^	Robert J. Harvey^54^	N/A
Mouse: B6.SJL-Slc6a3^tm1.1(cre)Bkmn^/J	The Jackson Laboratory	RRID:IMSR_JAX:006660
Mouse: B6;129S-Gt(ROSA)26Sor^tm32(CAG-COP4∗H134R/EYFP)Hze^/J	The Jackson Laboratory	RRID:IMSR_JAX:012569

**Oligonucleotides**		

Primers for RT-qPCR, see Table S4	This paper, Integrated DNA Technologies, Inc.	N/A

**Software and algorithms**		

PatchMaster	HEKA Elektronik GmbH	N/A
pClamp	Molecular Devices	N/A
Igor Pro 7.0.8.1	WaveMetrics, Inc.	N/A
ZEN 2009	Carl Zeiss Microscopy GmbH	N/A
ImageJ FIJI	Schindelin et al.^85^	http://fiji.sc/
Matlab R2021b	MathWorks	N/A
LSM Toolbox	Pirrotte et al.^86^	https://github.com/fiji/LSM_Toolbox
Bio-Formats plugin scripts	Linkert ^87^	https://github.com/ome/bioformats
qBasePlus software	Biogazelle	N/A
Ethovision XT	Noldus Information Technology BV	N/A
Graphic State 3.0 software	Coulbourn Instruments	N/A
Prism 8	GraphPad Software	N/A

**Other**		

Vibrating microtome (VT1200S)	Leica Microsystems IR GmbH	N/A
EPC-9 amplifier	HEKA Elektronik GmbH	N/A
CoolLED pE-2	CoolLED	N/A
ION-100 Iontophoresis Generator	Dagan Corporation	N/A
SF-77B Perfusion Fast-Step System	Warner Instruments	N/A
Zeiss LSM510 META	Carl Zeiss Microscopy GmbH	N/A
Plan-Apochromat 40x/0.95 Korr air objective	Carl Zeiss Microscopy GmbH	N/A
Argon laser	LASOS Lasertechnik GmbH	N/A
MaiTai DeepSee	Spectra-Physics Inc.	N/A
StepOnePlus Real-Time PCR System	Applied Biosystems	N/A
Automated operant chamber	Coulbourn Instruments	N/A
Open field (50 × 50 cm area with 30 cm walls; white)	BIOMED; UHasselt	N/A
T-maze (70 cm long, and 50 cm wide)	BIOMED; UHasselt	N/A

### Resource availability

#### Lead contact

Further information and requests should be directed to and will be fulfilled by the lead contact, Prof. Bert Brône (bert.brone@uhasselt.be).

#### Materials availability

This study did not generate new unique reagents.

### Experimental model and study participant details

#### Mice

All experiments were performed on adult male littermate mice (> 12 weeks). Male littermates with hemizygous presence (WT) or knockout (GlyRα2KO) of the Glra2 allele on a C57BL/6 background were used during all experiments.^53^^,^^88^

For the optogenetic studies, GlyRα2KO mice were crossbred with a DATIREScreAi32(RCL-ChR2(H134R)/EYFP) mouse strain on a C57BL/6 background. This triple transgenic mouse line with DAT-dependent expression of channelrhodopsin-2 (ChR2) allowed specific optogenetic stimulation of dopaminergic neurons in WT and GlyRα2KO animals. Male littermates heterozygous for DATIREScre and ChR2 allele, but hemizygous presence or absence of the Glra2 gene were used for experiments (respectively referred as WTs and GlyRα2KO mice in these experiments). The DATIREScreAi32(RCL-ChR2(H134R)/EYFP) mouse line was bred and was gifted by laboratory of Prof. M. Beckstead (used animal strains: JAX stock #006660 and JAX stock #012569).

Animals were maintained under a 12h/12h light/dark cycle with access to food and water *ad libitum* (except for the appetitive conditioning and T-maze experiments). Animal experiments approved by the local ethical committee at Hasselt University and in conformity with the EU directive 2010/63/EU on the protection of animals used for scientific purposes.

### Method details

#### Acute brain slice preparation

Acute brain slices were prepared for electrophysiological recordings and imaging experiments. Animals were cervically dislocated, and brains were rapidly isolated into oxygenated (95% O_2_ and 5% CO_2_ mixture) ice-cold cutting artificial cerebrospinal fluid (C-aCSF) containing (in mM): 140 choline chloride, 2.5 KCl, 1.25 NaH_2_PO_4_, 7 MgCl_2_, 26 NaHCO_3_, 0.5 CaCl_2_, 11.1 D-glucose. Sagittal slices (250 μm) were prepared on a vibrating microtome (VT1200S; Leica Microsystems IR GmbH, Wetzlar, Germany) and allowed a recovery of 1h at 36°C in oxygenated recovery solution (R-aCSF) containing (in mM): 127 NaCl, 2.5 KCl, 1.25 NaH_2_PO_4_, 3 MgCl_2_, 26 NaHCO_3_, 2 CaCl_2_, 11.1 D-glucose.

For electrophysiological measurements in the SNc oxygenated C-aCSF and R-aCSF contained (in mM): 127 NaCl, 2.5 KCl, 1.2 NaH_2_PO_4_, 1.2 MgCl_2_, 2.4 CaCl_2_, 21.4 NaHCO_3_, 11.1 glucose and 1.25 kynurenic acid. Horizontal slices of 200 μm were prepared of the ventral mesencephalon containing the SNc.

#### Electrophysiology

During recordings, slices were continuously perfused at a flow rate of 1.5-2 ml/min and maintained at a temperature of 36°C with oxygenated normal aCSF containing: 127 NaCl, 2.5 KCl, 1.25 NaH_2_PO_4_, 1 MgCl_2_, 26 NaHCO_3_, 2 CaCl_2_ and 11.1 D-glucose. Whole-cell recordings of dorsal SPNs were performed using borosilicate-glass (Hilgenberg GmbH, Malsfeld, Germany) pipettes with a resistance between 5-7 MΩ. All recordings were acquired with an EPC-9 amplifier (HEKA Elektronik GmbH, Lambrecht, Germany) and the PatchMaster interface (HEKA Elektronik). Recordings were acquired at a 20 kHz sampling interval and online filtered using a Bessel 2.9 kHz filter. Series resistances were checked before onset of whole-cell experiments and followed up regularly. If a change in series resistance exceeded 30%, the recording was discarded.

Visual and electrophysiological methods were used to identify SPNs^32^^,^^44^^,^^88^ in the striatum. Effects of DA modulation on intrinsic SPN excitability were recorded in whole-cell configuration using current clamp mode with an intracellular solution (290 mOsm) containing (in mM): 125 KMeSO_4_, 3 KCl, 0.022 CaCl_2_, 10 HEPES, 0.1 EGTA, 4 MgATP, 0.5 Na_2_GTP, 5 Na_2_phosphocreatine. Methanesulfonic acid was used to adjust pH to 7.2. Measurements were performed in the presence of 10 μM GABAA receptor blocker Gabazine, 100 nm GABAB receptor blocker CPG54626, 10 μM AMPA receptor blocker CNQX, 5 μM NMDA receptor blocker L-689,560, 0.1 μM nicotinic acetylcholine receptor blocker DHβE. A holding current was applied to keep SPNs at - 80 mV. The rheobase, *i.e.* the minimal current injection to reach action potential (AP) threshold, was determined by a current injection protocol using 10 pA current steps. AP frequency was measured at rheobase + 40 pA for 3 seconds and used as a reference baseline (BL) activity. After 20 seconds, a second rheobase + 40 pA current step was applied together with optogenetically induced DA release, using 470 nm light pulses (0.701 mW/mm^2^) of 4 ms at 20 Hz during 1 s. Here, current injections were performed to mimic SPN ‘upstate’ needed for positive activity modulation by D1Rs, while D2R-mediated SPN activity modulation is not affected by up- or ‘downstates’. After 5 minutes, the DA-modulated intrinsic SPN activity, measured as AP frequency, was measured at a rheobase + 40 pA injection combined with optogenetic stimulated DA release. Optogenetic pulses were generated by a CoolLED pE-2 (CoolLED, Andover, United Kingdom) illumination system triggered by an EPC-9 amplifier (HEKA Elektronik) and PatchMaster interface (HEKA Elektronik).

Dopaminergic neurons were visually identified as large neurons close to the medial terminal nucleus of the accessory optic tract (53). Electrophysiological properties of DA cells, *i.e.* spontaneous pacemaker firing (1–5 Hz) with wide extracellular waveforms (> 1.1 ms), were used to verify cell identification.^89^ Loose cell-attached recordings were performed to measure DA cell firing frequency. Recording pipettes (3-6 MΩ) contained a sodium-HEPES-based buffer (plus 20 mM NaCl; 290 mOsm/L; pH 7.35–7.40).^90^ Measurements were performed in the presence of 10 μM Gabazine, 100 nm CPG54626, 10 μM CNQX, 0.1 μM DHβE. Baseline pacemaking activity was recorded for 2 minutes. Sarcosine (500 μM) and glycine at low (30 μM) and high (1 mM) concentrations were bath perfused for 6 minutes to determine the effects of GlyR activation on pacemaking activity. Sarcosine, an inhibitor of the glycine transporter type-1, was co-applied during the different experiments to block the glycine reuptake out of the extracellular environment. Firing frequency analysis was performed on the last 2 minutes to allow glycine diffusion within the brain slice. Burst firing of DA cells was induced by NMDA iontophoresis (50 mM; pH 8.2; 500 ms pulse) using an ION-100 Iontophoresis Generator (Dagan Corporation, Minneapolis, United States) triggered by an EPC-9 amplifier (HEKA Elektronik) and PatchMaster interface (HEKA Elektronik). Iontophoretic pipettes with a resistance of 100–150 MΩ were used. D-serine (25 μM) was bath applied as a NMDA receptor co-agonist. To determine the effects of GlyRα2s on burst activity, glycine was bath-applied at low concentrations (30 μM) 3 minutes prior and during the experiment. Additionally, sarcosine (500 μM), a glycine transporter type 1 blocker, was co-applied to avoid the transport of low glycine levels out of the extracellular matrix. Glycine-induced chloride currents were recorded in whole-cell configuration voltage using voltage clamp mode. Recording pipettes (2.5–4 MΩ) contained (in mM): 57.5 K-gluconate, 20 NaCl, 57.5 KCl, 1.5 MgCl2, 10 HEPES, 0.025 EGTA, 2 MgATP, 4 Na_2_GTP. Glycine was applied for 10 s via a SF-77B Perfusion Fast-Step System (Warner Instruments, Hamden, CT, United States). Induction of phasic activity by optogenetic stimulation was verified in loose-cell attached configuration using the previously described protocol of striatal measurements.

Firing frequency recordings were analyzed using pClamp (Molecular Devices, LLC., San Jose, USA). Series resistance protocols and glycine-elicited currents were fitted and analyzed using Igor Pro 7.0.8.1 (WaveMetrics, Inc., Portland, United States).

#### Imaging

Expression of c-Fos, an immediate-early gene, in the striatum was measured 2 hours after D-amphetamine (5 mg/kg; i.p.) stimulation via immunofluorescence. Acute sagittal brain slices containing the striatum of WT and GlyRα2 KO mice were made as described above. Slices were fixed overnight in 4% paraformaldehyde (PFA) at 4°C and washed with Tris-buffered saline (TBS; 0.05 M Tris and 0.9% NaCl, pH 8.4) containing 0.05% Tween. Antigen retrieval was performed by incubating slices for 15 min in a citrate buffer (10mM tri-sodium citrate, 0.05% Tween 20, pH 6.0) at 95°C. Slices were cooled down on ice for 5 minutes followed by a blocking step (10% bovine serum albumin and 0.3% Triton X-100 in TBS) for 1 hour. Slices were then incubated with primary antibody rabbit anti-c-Fos (1:1000, Merck Millipore, Burlington, United States) in TBS (5% BSA, 0.3% Triton X-100) for 48h at 4°C. A secondary antibody labeled with Alexa Fluor 488 (goat, 1:500, Thermo Fisher Scientific, Waltham, United States) was applied for 90 min at room temperature. A nuclear counterstaining was performed using 4′,6-diamidino-2-phenylindole (DAPI) (Thermo Fisher Scientific) in TBS for 15 min. Slices were mounted in Fluoromount-G Mounting Medium (Thermo Fisher Scientific). Control immunostainings without primary antibodies were performed and showed no non-specific signals.

Microscopy images (Z-stacks) were acquired with a Zeiss LSM510 META (Carl Zeiss Microscopy GmbH, Jena, Germany) confocal microscope system mounted on an inverted Axiovert 200 M. A Plan-Apochromat 40×/0.95 Korr (Carl Zeiss Microscopy GmbH) air objective with cover slip adjustment (0.17) was used during image acquisition. A continuous wave Argon laser (488 nm; LASOS Lasertechnik GmbH, Jena, Germany) and a MaiTai DeepSee (790 nm; Spectra-Physics Inc., Santa Clara, USA) pulsed-laser were used to excite Alexa 488 and DAPI respectively. During the measurements laser power was kept low (5 mW at sample position) to limit the amount of photobleaching while collecting Z-Stacks. BP 500-550 IR and BP 390-465 IR emission filters were used. Images were acquired using the ZEN 2009 software (Carl Zeiss Microscopy GmbH). Z-stack tissue volumes contained typically 21 slices carrying dimensions of 225×225×42 μm. Pixel dwell time was 3.2 μsec. Limitations set by photobleaching required rapid collection of 2 μm step size Z-stacks over a depth of about 40 μm.

Processing and analysis of the acquired dual channel c-Fos and DAPI nuclear stain confocal image Z-stacks were carried out both with the open-source GNU General Public License image processing package ImageJ FIJI.^91^ and commercial Matlab R2021b (MathWorks, Eindhoven, The Netherlands). LSM Toolbox and Bio-Formats plugin scripts^85^^,^^86^ were used to read the data. DAPI stained Z-Stacks showed a very dense cell content. Specimen tissues carried a large variability dependent on treatment and control towards background and noise levels also regionally within individual optical slices. As reviewed and discussed elsewhere,^87^^,^^92^^,^^93^ these observations in combination with the increased scattering and weaker signals from deeper layers in the Z-Stacks made automatic segmentation of the c-Fos channel a challenge. To minimize an undercounting bias of c-Fos positive cells that can amount to 50% in the deeper tissue layers of the raw Z-stacks, due to intensity decay, images were normalized considering the histograms of the individual slices in combination with a Z-stack normalization near the mean (optical slice 11) of the Z-stack as indicated by.^94^ Effects of uneven illumination were reduced via a generalized neighborhood non-local means denoising plugin.^95^^,^^96^^,^^97^ Reduction of the influence of locally varying overall background via a rolling ball approach had limited success. Subsequently for optimization and validation during interactive morphological segmentation^98^ enhanced 2D Z-Projection color images - showing the outlines of brighter c-Fos objects discernible above the noisy background - were simultaneously displayed side-by-side with the Z-projections of the binary corrected Z-Stacks. Operator supervised iterative adjustment of automated thresholding proved most effective and least time-consuming. Segmentation quality was validated by expert ground truth verification. Cell counting was performed manually on the 2D Z-projections. Z-stacks were visually checked throughout all optical slices.

#### RT-qPCR

Relative c-Fos gene expression level, relevant to dopamine-modulated striatal activity, was measured 2 hours after d-amphetamine (5 mg/kg; i.p.) stimulated and saline control animals. The striatum was dissected from WT and GlyRα2KO mice and homogenized. For the quantification of the GlyR subunit expression in the midbrain, the SNc was dissected and homogenized. Total RNA was isolated by the guanidinium thiocyanate-phenol-chloroform procedure using QIAzol lysis reagent (Qiagen, Hilden, Germany) followed by RNeasy Kit (Qiagen) purification. RNA purity and quantity were checked by NanoDrop 1000 Spectrophotometer (Thermo Fisher Scientific). Purified RNA was converted to cDNA using the High-Capacity cDNA Reverse Transcription Kit (Applied Biosystems, Foster City, United States). RT-qPCR was performed in a Fast SYBR Green Master Mix (Applied Biosystems) containing 12.5 ng cDNA template, 3 mM forward and 3 mM reverse oligonucleotide primers (Integrated DNA Technologies, Inc., Leuven, Belgium), and RNAse-free water was added to a total reaction volume of 10 μl.

Expression levels of target genes were normalized against the most stable housekeeping genes, Gadph and Hprt, determined by qBasePlus software (Biogazelle, Ghent, Belgium). Comparative threshold cycle (Ct) quantitation (59) was performed using a StepOnePlus Real-Time PCR System (Applied Biosystems). Expression levels were converted to fold changes against the WT mean value. PCR product specificity was evaluated by melting curve analysis (StepOne Software V2.3; Applied Biosystems). Primer efficiencies between 95-105% were checked using standard curves of pooled samples and equation: E = 10[–1/slope].

#### Behavior

All animals were handled daily for two weeks prior to the start of the experiments. During all tests, animals were randomized for their genotypes and/or treatment.

#### Circadian activity

Circadian locomotor activity was monitored for 24 hours in individually housed mice in standard home cages and measured as the number of crossings of infrared beams (3/cage), which were connected to a PC-operated counter.

#### Psychostimulant-induced locomotor activity

Locomotor activity was video recorded within an open field (50x50 cm; 30 cm walls). Baseline activity was measured for 1 hour followed by a d-amphetamine (5 mg/kg; i.p.) or cocaine (20 mg/kg; i.p.) injection to measure dopamine-stimulated locomotion for another 2 hours. Total distance travelled was processed and analyzed in 10 min bins using the Ethovision XT (Noldus Information Technology BV, Wageningen, The Netherlands) software.

#### Appetitive conditioning

Appetitive conditioning was conducted in 16 identical automated operant chambers (Coulbourn Instruments, Allentown, PA), each set in a ventilated, sound-isolated cubicle (Coulbourn Instruments, Allentown, PA). Test cages were equipped with a grid floor connected to an electric shocker, a pellet feeder, and a nose poke operandum. Mice (n = 8/group, except control: n = 7) were placed on a food restriction schedule, and kept at 80–90% of their free-feeding weight. Mice were trained in daily trials of 30 min during which they learned to use a nose poking device to obtain food pellets (Noyes precision pellets, Research Diets, New Brunswick, NJ). Mice received food pellets during all trials, but the reinforcement schedule was gradually increased to obtain a stable response rate. Rate of nose poking in each trial was recorded with Graphic State 3.0 software (Coulbourn Instruments, Allentown, PA). Training started with one continuous reinforcement with additional guaranteed pellet delivery every 120 s (CRF + 120), followed by CRF (every nose poke rewarded), fixed ratio trials (FR5, reward on every 5th nose poke; FR10, reward on every 10th nose poke), and variable ratio trials (VR10, reward on average every 10th nose poke). Once a group of animals was stably poking (defined as no effect of time using a one-way ANOVA) for three consecutive trials, the group was moved to the next reward schedule. Transition between reward schedules happened after three days of stable poking. During six extinction trials, animals no longer received pellet rewards. On the reinstatement trial, animals were placed back on a VR10 reward schedule.

#### T-maze

The T-maze was 70 cm long, and 50 cm wide. Before training mice were allowed to explore the T-maze for ten minutes on three consecutive days. Animals were placed on a food restriction schedule, and kept at 80-90% of their free-feeding weight. Animals were then tested on six consecutive days, eight trials per day. A cup with ten food pellets was placed in the rewarded arm. In the unrewarded arm, ten pellets were placed as well to avoid odor confounds, but these were not accessible to the animal. Visual cues were placed on the end of the arms. The cue that was associated with the reward was randomized over the animals, and the location of the reward (left arm or right arm), was randomized over trials within animals. At the start of the trials, animals were placed in a start box. After 5 seconds, the start box was opened by manually lifting a divider. The latency to reach the intersection (entry with center of body) was measured and used as a measure for motor activity. Once an animal left the decision box (dark grey area in Figure 4D) divider was inserted to prevent the animals from leaving the arm of choice. Time to consume the reward was scored manually, and latency to start consuming the reward once a correct choice had been made (i.e. from the time the mouse left the decision box) was used as a measure of motivation. The number of pellets consumed was recorded for each trial as well and used as a measure for the hedonic response.

#### Anxiety and stress response

Anxiety and stress response were measured in an open field (50x50cm). Mice were placed in the arena for 1 hour and their run tracks were recorded and analyzed using the Ethovision XT (Noldus Information Technology BV) software. Eight hours after the behavioral experiment, faeces were collected as described by.^99^ Briefly, mice were individually housed in cages with a grid floor that allowed for faeces to drop through. Filter paper was placed on the bottom to absorb urine. Mice were habituated to the grid floors the three days before testing. Fecal corticosterone metabolites were analyzed. Briefly, each fecal sample was homogenized and an aliquot of 0.05 g was shaken with 1ml of 80% methanol for 30 min on a multi vortex. After centrifugation, an aliquot of the supernatant was diluted (1:10) with assay buffer and frozen at -20°C. To determine the amount of fecal corticosterone metabolites, we used a 5α-pregnane-3β,11β,21-triol-20-one EIA, which utilizes a group-specific antibody measuring steroids with a 5a-3β,11β-diol structure.^99^ Since steroid excretion is affected by the activity of the animals, we performed this experiment in a reversed light-dark schedule (6-18 lights off). However, the arena was lit from beneath. The experiment was repeated 7 and 14 days later.

### Quantification and statistical analysis

Statistical analysis was performed using Prism 8 (GraphPad Software, San Diego, United States). Data were checked for normality and differences in variance. Sample sizes are represented in the legend of the corresponding figure. For the electrophysiological experiments the sample size represents the number of cells from at least three different animals. The sample size of the behavioral studies represents the number of animals. Statistical significance was defined as followed: ∗p < 0.05; ∗∗p < 0.01; ∗∗∗p < 0.005. A detailed overview of the performed statistical analyses with post-hoc tests can be found in Tables S1 and S2. All experiments were performed in a blinded and randomized manner until data analysis.

## Data Availability

- All data is represented in the main paper and/or supplemental information. The software, algorithms and codes which are used in the paper are referred to in the key resources table. - The present study did not produce original code. Additional information on the reported data is available from the lead contact upon request. - Any additional information required to reanalyze the data reported in this paper is available from the lead contact upon request.

## Supplemental information

Document S1. Figures S1–S3 and Tables S2–S4

Table S1. Statistical analyses details, related to Figures 1–4
