# Supplementary material for: Dopamine-mediated striatal activity and function is enhanced in GlyRα2 knockout animals
Source: iScience. 2023 Jul 17;26(8):107400. doi: 10.1016/j.isci.2023.107400 (PMC10404725; doi:10.1016/j.isci.2023.107400)
Supplement: Document S1. Figures S1–S3 and Tables S2–S4 [file mmc1.pdf]

iScience, Volume *26*

Supplemental information

Dopamine-mediated striatal activity and function is enhanced in GlyRα2 knockout animals

Jens Devoght, Joris Comhair, Giovanni Morelli, Jean-Michel Rigo, Rudi D'Hooge, Chadi Touma, Rupert Palme, Ilse Dewachter, Martin vandeVen, Robert J. Harvey, Serge N. Schiffmann, Elisabeth Piccart, and Bert Brône

Supplemental information titles and legends

Figure S1, related to Figure 2. Optogenetic stimulation of dopamine neurons. (A) Optogenetic stimulation (10 pulses, 20 Hz) of channelrhodopsin- 2-expressing dopamine cells in the substantia nigra pars compacta induces burst activity. (B) Control SPN recordings in which DA terminals were not stimulated optogenetically do not show a significant change in action potential frequency over time.

Figure S2, related to Figure 2. **Baseline dopamine neuron firing is unaltrered in GlyRα2 Kos.** (A) Inter-spike interval during pacemaking activity of dopamine neurons is unaltered in GlyRα2 KO mice. (B) The mean inter-spike interval of a burst in dopamine cells is unaltered in GlyRα2 KOs compared to WT littermates. (C) Dopamine cells from GlyRα2 and WT littermates fire equally regularly during an NMDA-induced burst. (D) Strychnine application does not alter the pacemaking activity of dopamine cells in WT mice (represented as 1 min bins). (E) Exemplar traces of dopamine neuron pacemaking firing without (left) and with strychnine application (right) in WT mice. (F) Baseline firing rate of dopamine neurons is unaltered in GlyRα2 KO animals compared to WT littermates in the absence of $GABA_AR$ and $GABA_BR$ blockers. Moreover, there is no change in baseline firing rate in response to 30 µM glycine and sarcosine (500 µM) in either GlyRα2 KO or WT mice. (G) Exemplar traces of pacemaking firing in response to 30 µM glycine and sarcosine (500 µM), and absence of $GABA_AR$ and $GABA_BR$ blockers in WT (top) and GlyRα2 KO mice (bottom). Data are represented as mean ± SEM.

Figure S3, related to figure 4. **GlyRα2 KOs show unaltered baseline locomotor activity and response to cocaine administration.** (A) GlyRα2 KO animals exhibit no changes in circadian activity compared to WT littermates. (B) The locomotor response to 20 mg/kg cocaine (i.p.) is unaltered in GlyRα2 KO animals compared to WT littermates.

Table S2. Membrane resistance is unaltered in GlyRα2 knockout mice, related to Figure 1.

Table S3. Raw data histograms, related to Figure 3.

Table S4. Primer sequences, related to Figures 1-4.

Figure S1

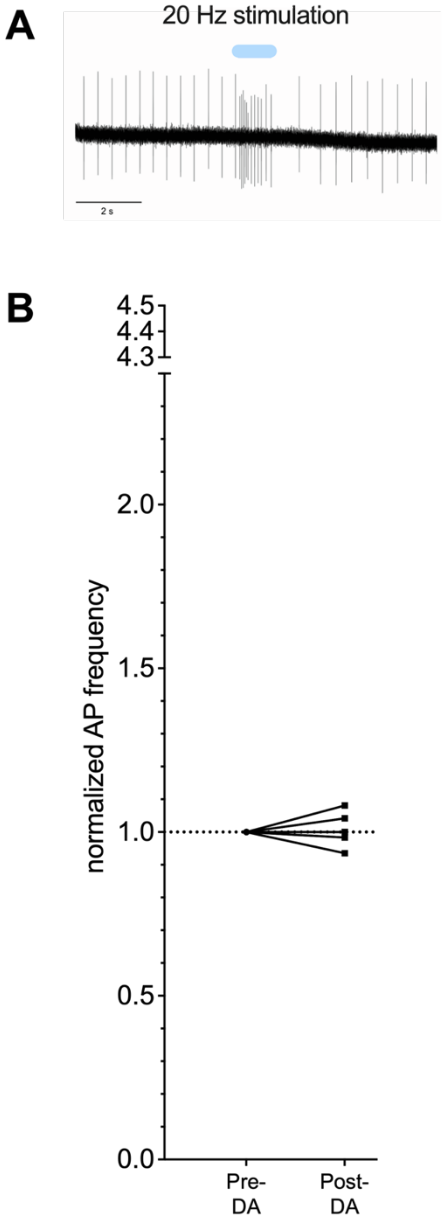

A
20 Hz stimulation
2 s
B
normalized AP frequency
4.5
4.4
4.3
2.0
1.5
1.0
0.5
0.0
Pre-
DA
Post-
DA

Figure S2

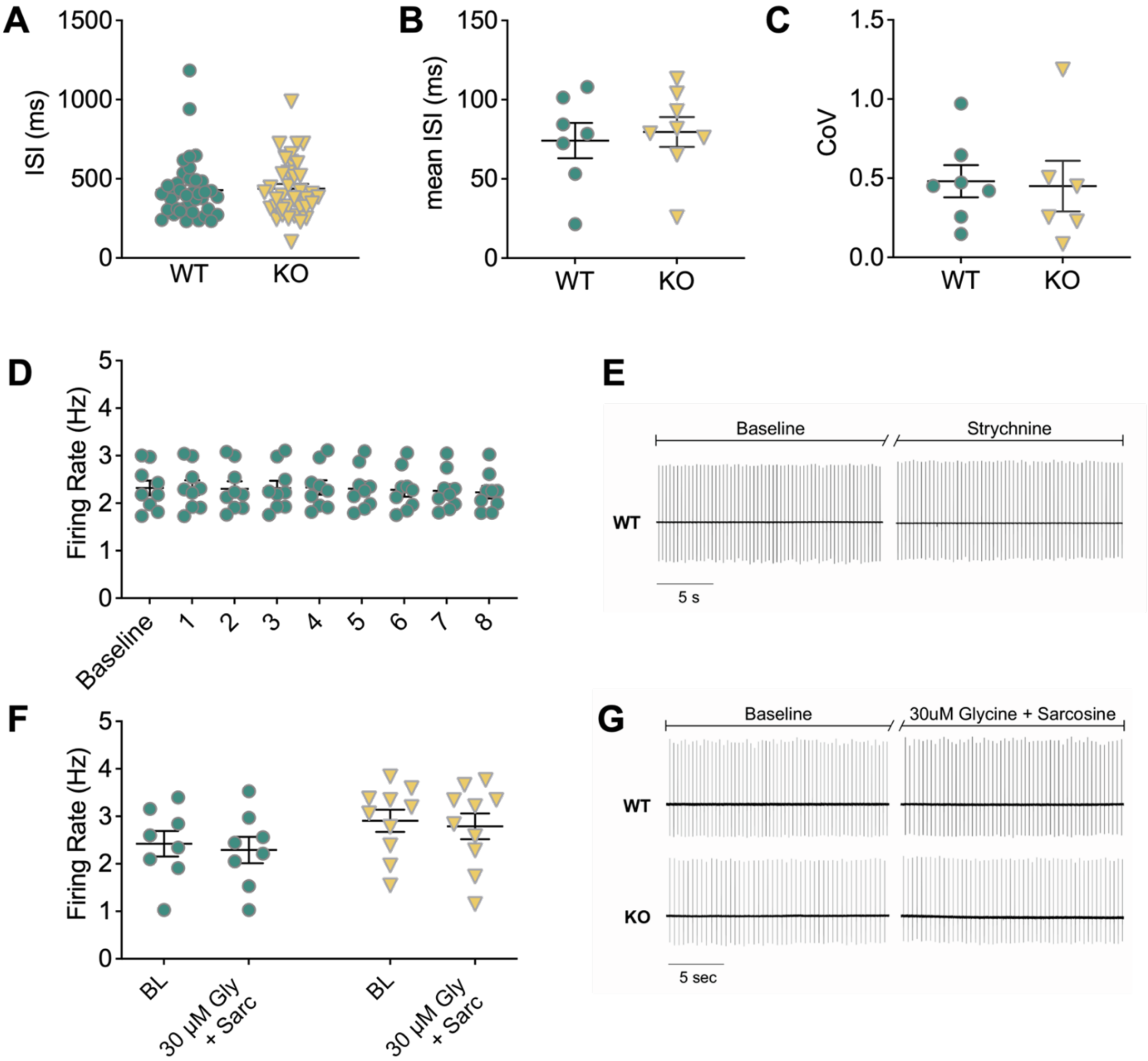

A
ISI (ms)
1500
1000
500
0
WT
KO
B
mean ISI (ms)
150
100
50
0
WT
KO
C
CoV
1.5
1.0
0.5
0.0
WT
KO
D
Firing Rate (Hz)
5
4
3
2
1
0
Baseline
1
2
3
4
5
6
7
8
E
Baseline
Strychnine
WT
5 s
F
Firing Rate (Hz)
5
4
3
2
1
0
BL
30 µM Gly + Sarc
BL
30 µM Gly + Sarc
G
Baseline
30uM Glycine + Sarcosine
WT
KO
5 sec

Figure S3

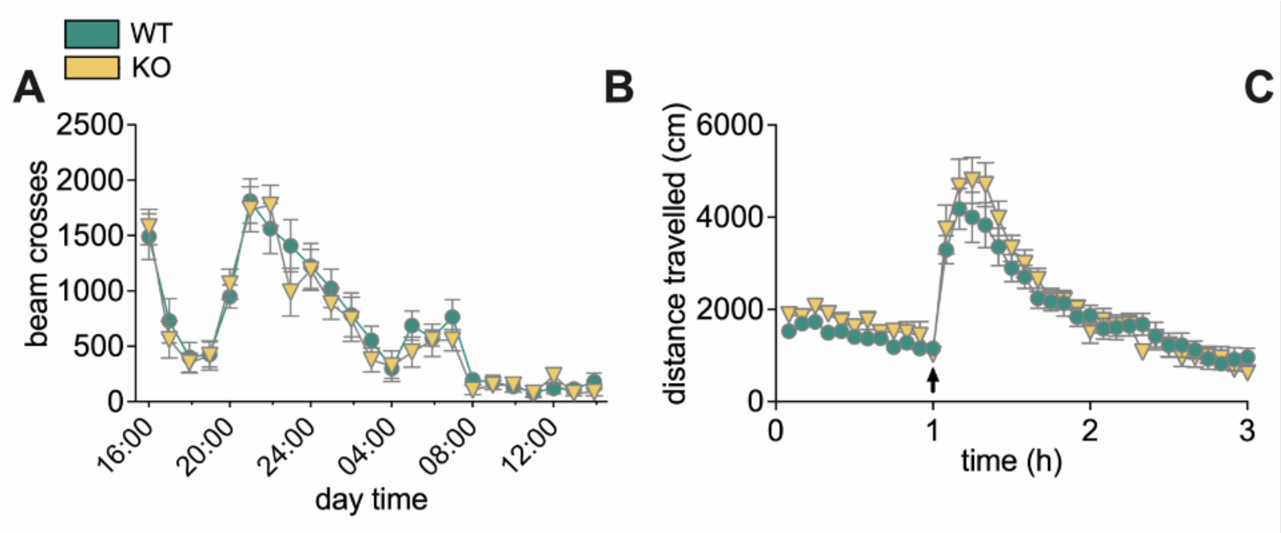
WT
KO
A
B
C
beam crosses
2500
2000
1500
1000
500
0
16:00
20:00
24:00
04:00
08:00
12:00
day time
distance travelled (cm)
6000
4000
2000
0
0
1
2
3
time (h)

Table S2

Membrane resistance			
	**WT (Mean ± SEM)**	**GlyRα2 KO (Mean ± SEM)**	**Statistical test**
**p-D1 SPNs**	55.8 ± 8.2 MOhm	41.8 ± 2.6 MOhm	Mann-Whitney<br>U = 13, p = 0.18
**p-D2 SPNs**	42.7 ± 4.7 MOhm	61.5 ± 6.7 MOhm	Mann-Whitney<br>U = 29, p = 0.07

Table S3

WT			
**Nr**	**Amplitude (frequency)**	**Peak (distance in um)**	**Width (um)**
**1**	4.9624	12.0753	5.8300
**2**	10.0365	16.1598	5.8300
**3**	9.6124	18.6553	1.1209
**4**	5.2098	21.4277	2.6007
**5**	5.6347	27.0647	3.6959
**6**	4.2599	32.3122	2.2850
**7**	5.6453	36.2405	1.5019
**KO**			
**Nr**	**Amplitude (frequency)**	**Peak (distance in um)**	**Width (um)**
**1**	17.6486	12.9034	3.9717
**2**	19.1571	17.8990	3.1405
**3**	21.6507	20.6581	2.0895
**4**	18.4631	23.6752	3.2088
**5**	17.6675	28.6784	3.5434
**6**	9.3143	32.4364	1.7477

**7**	11.6588	35.6434	1.4952

Table S4

Primer sequences		
**Gene**	**Forward**	**Reverse**
**Glra1**	ATCACAAGAGCCCCATGCTAAA	TGTTGTTGTTGTTGGCACCC
**Glra2**	CACTGGCAAGTTTACCTGCAT	GGAGACCCAGGACAAAATGA
**Glra3**	GACGGAAGCTTTTGCACTGG	GACAGGGCCCCATTCCATAG
**Glra4**	CAGCATCAGATTGACCCTCA	GCAGGAGCATCTTCTAGCCA
**Glrb**	TGAGCTGCTGAAACTTCCGT	CTCGGGTGACTGCTGAGATG
**Gapdh**	ACCACAGTCCATGCCATCAC	TCCACCACCCTGTTGCTGTA
**Hprt**	CTCATGGACTGATTATGGACAGGAC	GCAGGTCAGCAAAGAACTTATAGCC
